# Policy approaches to health system performance assessment: a call for papers

**DOI:** 10.2471/BLT.23.290288

**Published:** 2023-07-01

**Authors:** Irene Papanicolas, Dheepa Rajan, Marina Karanikolos, Dimitra Panteli, Kira Koch, Josep Figueras

**Affiliations:** a Center for Comparative Health Systems, Brown University, Providence, United States of America.; b European Observatory on Health Systems and Policies, World Health Organization, Place Victor Horta 40, 1060 Brussels, Belgium.; c European Observatory on Health Systems and Policies, World Health Organization, London, England.; d Special Programme for Primary Health Care, World Health Organization, Geneva, Switzerland.

Health system strengthening is key to achieving universal health coverage, a target of the sustainable development goals. For policy-makers to effectively focus health system strengthening efforts and translate them into improved health system performance, they need to determine which areas should be prioritized and receive resources. Regular health systems monitoring, appraisal and assessment are important to better comprehend the key strengths and shortcomings of health systems.

Assessing health system performance requires understanding all components of a health system, as well as its boundaries. Over the last two decades, several conceptual health system frameworks have been produced to help in understanding health systems,^1^^–^^5^ many of which have also supported efforts to measure health system performance. In parallel, several health system assessment tools have been created to provide a system-wide and comprehensive analysis of relevant health system areas such as health financing, governance, human resources for health and health-care programmes.^6^^–^^11^ All of these efforts aim to provide a clear and simple common conceptualization of the health system to facilitate further progress in achieving health policy goals.

These approaches seek to support policy-makers in describing the current structure of the health system, such as through the health system functions or building blocks, and in evaluating these structures in light of health system reform. Some assessment approaches focus on understanding how well the system is performing overall by examining the extent to which health systems meet a set of defined objectives. These approaches usually rely on quantitative metrics and analytical methods, often referred to as health system performance assessment. Ultimately, health system strengthening relies both on assessing health system functions and on attaining system goals, while clarifying the relationship between the two.^12^

Key policy concerns in a variety of settings can then be turned into action through analysis of the following considerations. First, how does the pooling of resources influence access, quality and financial protection? Second, how do different models of service delivery, such as one more oriented towards primary health care, influence how people-centred and efficient the health system is? Third, how resilient are health system functions and health system outcomes to specific external shocks?

The *Bulletin of the World Health Organization* will publish a theme issue on health system performance assessment, which aims to throw a spotlight on such policy concerns. Manuscripts should aim to showcase how health system performance assessment can answer these policy considerations, as health systems are rebuilding after the coronavirus disease 2019 (COVID-19) pandemic, and reorienting their systems towards stronger primary health care and better emergency preparedness.

This theme issue aims to show our current evidence and knowledge base in terms of real-world applications of performance assessments, emphasizing recent developments in the collection and linkage of data on health system structures, resources and expenditures with service delivery and outcomes. The pandemic prompted the need for combining quantitative data sets and qualitative social science information, often in real time, to facilitate decisions on crisis measures. To foster sustainable recovery and resilience to future shocks, we must better understand how factors within and outside the health system affected health system goals, such as health improvement, equity and access. As policy-makers seek to ensure that health systems can respond to constituents’ needs in a timely and adequate manner, taking stock of the improvements in global data collection and availability, and new methods and approaches to health system performance assessment, is important.

This theme issue seeks to contribute to these aims and to the evidence base for health system strengthening. We welcome papers that expand our current knowledge by examining how different countries and settings, with their unique health system structures, contexts and policies, have applied different health system performance assessment approaches; addressed the challenge of data gaps; tried and tested innovative data collection approaches; and used data to understand health system strengths and weaknesses to ultimately improve system performance and health policy implementation.

Papers may focus on policy-relevant areas of health system structures or functions that so far have proven difficult to evaluate, such as health systems governance or integrated care. Examples of health systems where specific policies or structural redesign have been undertaken to improve health system performance are of special interest. Studies focused on health system comparisons or those that track evolution in health system performance over time are also welcome. The deadline for submissions is 15 November 2023. Manuscripts should be submitted in accordance with the *Bulletin’s* guidelines for contributors (available at: https://www.who.int/publications/journals/bulletin/contributors/guidelines-for-contributors) and the cover letter should mention this call for papers.
